# Effects of Nature-Based Group Art Therapy Programs on Stress, Self-Esteem and Changes in Electroencephalogram (EEG) in Non-Disabled Siblings of Children with Disabilities

**DOI:** 10.3390/ijerph18115912

**Published:** 2021-05-31

**Authors:** Soo-Ji Kang, Hyon-Suh Kim, Kwang-Hyun Baek

**Affiliations:** 1 Department of Medicine, CHA University, Seongnam-si 13488, Gyeonggi-do, Korea; speedrabbit823@gmail.com; 2 Ewha Child & Youth Development Center, Ansan-si 15541, Gyeonggi-do, Korea; hyonyoung2@hotmail.com; 3 Department of Biomedical Science, CHA University, Seongnam-si 13488, Gyeonggi-do, Korea

**Keywords:** art therapy, forest therapy, disability, EEG, stress, natural environment

## Abstract

The purpose of the present study was to examine changes in brain waves, stress, and self-esteem after a continuous eight-week nature-based art therapy program in the forest in non-disabled siblings of children with disabilities. A total of 29 participants participated in this study (art therapy program group, *n* = 18; control group, *n* = 11). The art therapy program group received eight weekly sessions of art therapy lasting 60 min each. Pre- and post-test results showed positive changes in the brain function index and stress levels of the participants in the art therapy program group. On the self-esteem scale, overall and social self-esteem increased significantly. In conclusion, creative activities in the forest can increase resistance to diseases through mechanisms that relieve stress and increase self-esteem. If art therapy that emphasizes somatosensory experience, creative expression, and self-motivation is accompanied by forest activities, this combined intervention can elicit positive physical and psychological changes.

## 1. Introduction

The illness or disability of one member of the family affects the relationships among the rest. However, this does not mean that families having a child with a disability or their siblings are less adjusted [1]. In general, studies show that there are cases in which siblings of children with disabilities adjust better than comparable subjects without such experiences [2,3,4]. However, many studies have reported that emotional and behavioral difficulties increase among siblings of children with disabilities [5,6,7,8,9,10,11].

Non-disabled siblings of a disabled child naturally have both positive and negative experiences regarding their sibling situation. In addition to the roles expected in general sibling relationships, non-disabled siblings in families with children with disabilities may protect children with disabilities, act as a proxy for their parents, and play a major role in socialization by becoming playmates for children with disabilities [12,13]. Some have excessive responsibilities, while others feel an enormous pressure to be achievers because parents’ psychology of compensation places disproportionate expectations on their non-disabled children [14]. Such pressure may be stressful for these children, possibly reducing their self-esteem. This stress that children feel is closely related to physical illness and overall mental health, thereby affecting holistic growth [15,16,17,18,19]. Therefore, such stress must be recognized early and appropriately managed. The most crucial internal resource for children to adapt to stressful situations is self-esteem. Self-esteem is protective, helping them overcome stress [20]. It is an essential factor in an individual’s healthy adaptation to the environment, personal development, and positive self-realization [21]. Instilling high self-esteem in children is a central task in their development, particularly in adolescents. It reduces problematic behavior in students, while low self-esteem increases stress [22,23].

### Nature-Based Group Art Therapy

According to the American Art Therapy Association, art therapy facilitated by a professional art therapist effectively supports personal and relational treatment goals as well as community concerns [24]. Art therapy is used to improve cognitive and sensorimotor functions, foster self-esteem and self-awareness, cultivate emotional resilience, promote insight, enhance social skills, reduce and resolve conflicts and distress, and advance societal and ecological change [24]. In particular, art therapy with balanced physical and emotional healing helps the body and mind work harmoniously. In addition, art therapy allows individuals to project their problems or emotions onto external objects, thus gaining others’ empathy with the image.

In this study, the concept of nature-based art therapy was defined by a fusion of nature therapy and group art therapy, which are based on activities in nature. In addition, nature-based group art therapy, as used in this study, was intended to alleviate human physiological and emotional conflict through image representation and exert preventive and restorative effects. Nature-based group art therapy can further shorten the process of building trust among group members by maximizing the use of additional elements, such as sound, wind, and smell, provided in the forest environment [25]. In group activities, participants can exchange emotions and thoughts, listen to each other, and become interested in others’ creations; these allow relationship building [26]. Group art therapy aims to improve problem solving through art and help individuals who encounter new environments [27]. Therapy in the natural setting expands the classical psychological and psychodynamic approach [28,29]. The process of experiencing nature through art therapy helps people connect with the healing elements that exist in nature [28,30]. Nature can be a healing space for children to find psychological and social stability [31]. Art therapy related to forest activities includes and supports experiential learning and play and focuses on physical and sensory experiences, creative expression, and spontaneity in natural environments [32,33,34].

Although many previous studies have focused on the therapeutic function of forests, most have involved one-off sessions rather than a continuous program. For example, one study that ran a forest healing camp for 3 days and 2 nights for individuals with depression showed that the participants had more alpha (α) waves, known as stable waves, after attending the camp [35]. The forest also has physiological effects, such as calming frontal lobe functions and lowering cortisol levels, both of which are associated with stress [36]. Furthermore, forest therapy utilizes various elements of nature, such as fragrance and scenery, to increase immunity, promote health, reduce stress, and improve mental health, self-esteem, confidence, and subjective well-being [37,38,39,40]. In addition, students’ self-efficacy, self-confidence, and peer relations improved with their academic abilities, and depression was reduced, suggesting that the therapy improved social relationships both quantitatively and qualitatively [36,41,42].

This study aimed to examine changes in brain waves, stress, and self-esteem after a continuous 8-week nature-based art therapy program in the forest for non-disabled siblings of children with disabilities.

## 2. Materials and Methods

### 2.1. Methods and Experimental Design

This study was conducted from October 2018 to October 2019 on Suri Mountain, Gwacheon, in South Korea. A safe and well-managed forest area was selected for the study. Participants were recruited by posting a recruitment notice at the counseling center for children with disabilities from October 2018 to April 2019.

The criteria for study participants are as follows:

- Non-disabled siblings of children with disabilities.
- Children aged 7–13 years old.
- Children who can obtain parental consent to participate in the study.
- Children who can be accompanied by their parents to the study site.
- Children without art therapy experience.

The participants were randomly divided into experimental and control groups. Randomization was performed using simple randomization and was developed by college student volunteers who were not involved in the study intervention. The volunteers created a random assignment table and assigned participants to the experimental and control groups. This was a single-blind study.

A total of 36 participants were recruited in the study (control group, 18 participants and experimental group, 18 participants), of which 33 consented (92% consent rate) and three participants were excluded (e.g., do not meet the criteria for study participants). Finally, 33 participants were randomized (15 and 18 participants in the control and experimental groups, respectively); four participants dropped out due to personal reasons (e.g., it became difficult for parents to accompany their children to the study site). Thus, 11 participants from the control group and 18 participants from the experimental group completed the study.

The study period was 8 weeks, during which 60-minute group art therapy sessions were conducted every week. One art therapy supervisor evaluated the methods and scales used for the program. The experimental group underwent art therapy in the forest area, whereas the control group was free to remain active outside the said area. This study was conducted in the form of group art therapy rather than a psychological education program. Art therapy in nature was introduced to the participants as a time to explore nature with friends, create creative work, and communicate through works.

### 2.2. Sample Size

The sample size was calculated using the sample size calculator by setting the population as the total number of recruits, the confidence level of 95%, and the standard error of 5%; the required sample size was 33 participants in total.

### 2.3. Simulation

The first planned period and program before the start of the study were to conduct 16 art therapy programs twice a week for 8 weeks. We visited several forests and chose two forests that are safe and suitable for children to explore. To effectively perform art therapy in the forest, art therapists participating in the study simulated 16 art therapy programs in the two forests for 2 months (Table S1). The simulation was conducted as a concept to see the analysis of the progress of art therapy programs and the progress time, and it was not a process for deriving statistical validity results. During the program simulation, art therapists explored the forest terrain and natural objects that could be used during the program.

Through the simulation, the program progression method and the program methodology were modified. It was expected that it might be difficult for the parents of the participants to accompany them to the research site twice a week to take part in the program. In addition, it was difficult to run the program on a set date and time due to weather changes, such as fine dust and heavy rain. Through the simulation, the program was modified to run once a week for 8 weeks for 60 min. In addition, eight sessions were selected as suitable programs for group therapy, allowing more use of nature. One of the two forests was selected as a suitable location for the study. The selected location had the following characteristics: the mountain slope is not too steep, there are many kinds of trees and plants, there is drinking water, there are toilets that can be used, and it has flat land and valleys.

### 2.4. Intervention

The art therapy program was conducted by one clinical art therapist and one assistant therapist. The main therapist has a Ph.D. in clinical art therapy and is an art therapy expert with over 7 years of experience. The assistant therapist has a master’s degree in clinical art therapy and over 3 years of experience.

Participants in the experimental group received basic forest safety training before the program started. Forest safety education aimed to introduce safety rules that will help participants in the case of dangerous situations in the forest. For example, how to climb a mountain, tend to an ankle injury, wounds, or an insect bite, and how to deal with dangerous situations were explained in advance.

For 10 min after starting the session, the participants were asked to find a place on the mountain where they could work. Once they found a place, they were instructed to find materials from their surroundings to create an artwork for 40 min. For the remaining 10–15 min, the participants discussed their projects. The participants were free to express their opinions and use materials from nature. During the program, art therapists explained the aims of the program and acted as a mediator in coordinating the opinions of participants.

In this study, we attempt to induce an ecological approach using natural objects provided in the forest as art therapy materials. In addition, it is composed of an art therapy program that allows them to experience psychological relaxation and recovery by using various stimulating factors that can be experienced in the forest by taking advantage of the forest environment. The art therapy comprised eight sessions, and it is described in Table 1.

### 2.5. Approval and Accordance

This study was approved by the CHA University Institutional Review Board in October 2018 (1044308-201809-HR-051-03). Participants were children aged 7–13. Therefore, the details of the program were explained to the participant’s parents, and parental written consent was obtained. In addition, participants were also given a basic explanation of the program. The consent form outlined the purpose, process, method, duration, side effects or risks, benefits, and disadvantages of the study. In addition, the confidentiality of the personal information collected was guaranteed.

### 2.6. Measures

Electroencephalogram (EEG): EEG was measured using the Neurofeedback System (Panaxtox Corp, Seoul, Korea) developed in the Korea Psychiatry Research Center. This device measures the left and right EEG traces simultaneously in FP1 and FP2 of the prefrontal lobe using two electrodes in a sequential bipolar montage, as defined by the International 10–20 system. A solid electrode is attached to the headband, and the EEG is measured from the left and right frontal lobes through the FP1, FPz, and FP2 channels, fixed at 4 cm intervals on the left ear lobe as a round electrode. EEG measurement can assess real-time brain function and provides easy access to objective brain function analysis. EEG is a frequency-based spectrum analysis method that captures the degree of slow and fast waves by correlation and provides more information than the existing independent analysis method for each [43]. Therefore, the brain function index can be ascertained through EEG analysis (Table 2).

Attention quotient (ATQ): The ATQ represents the degree of brain arousal and resistance to disease or stress. It is calculated by dividing the activity of theta (θ) waves by that of sensorimotor rhythm (SMR) waves of around 12–15 Hz. Thus, it is related to immunity against disease or stress [43,44].

Anti-stress quotient (ASQ): ASQ represents the physical and mental fatigue caused by internal and external environmental factors. ASQ can be calculated by correlating delta (δ) waves with high beta (β) waves. Thus, it is a measure of resistance to stress [44,45].

Stress scale: Han’s (1996) stress scale, a modified scale adapted for children based on the “Daily Hassles Questionnaire” devised by Rowlison and Felner (1988), was used. In this study, the 36-item questionnaire consisted of five sub-elements: parents, family environment, friends, academics, and school [46,47].

Self-esteem scale: The self-esteem scale was developed by Choi and Jeon (1993) for diagnosing children and adolescents [48]. The scale consists of 32 questions in four sub-domains (overall self-esteem, social self-esteem, self-esteem at home, and self-esteem at school).

### 2.7. Data Collection and Procedures

Both groups underwent a pre-test before the first session and a post-test after the last session. Pre- and post-tests were performed individually in a laboratory within the Child Development Center to control external conditions. The EEG test time was conducted for 10 min, including the preparation time, and stress and self-esteem scales were conducted for 40 min. Participants were assigned a numerical number, and pre- and post-data were managed and documented with the personal numbers assigned to the participants.

### 2.8. Statistical Analysis

Statistical analyses were conducted using SPSS statistics (version 22.0). All data were checked for normal distribution using the Shapiro-Wilk test, Q-Q plot, and histogram. Specifically, frequency analysis was performed on the participants’ sociodemographic characteristics. An independent two-sample *t*-test was performed to ascertain whether demographic characteristics, EEG, stress, and self-esteem levels were homogeneous in the experimental and control groups. In addition, a paired t-test was conducted to examine the effects of art therapy on brain waves, stress, and self-esteem. The significance level was set at α = 0.05.

## 3. Results

### 3.1. Group homogeneity Test and Frequency Analysis

The general characteristics of the participants are shown in Table 3. The average age was 8.6 years; 18 participants (62.1%) were male, and 11 (37.9%) were female. All participants were non-disabled, with no compromising diagnosis, and were siblings of children with disabilities. Table 4 shows the results of the groups’ homogeneity test before the program. The pre-test results identified sex, age, stress level, and self-esteem to be homogeneous.

### 3.2. Effects of Nature-Based Group Art Therapy on Brain Waves, Stress, and Self-Esteem

As shown in Table 5, clinical art therapy positively affected the changes in brain waves in the experimental group compared to the control group. In the experimental group, changes were observed in the left (*t* = −2.741, *p* < 0.05) and right (*t* = −2.256, *p* < 0.05) brains on ASQ, and positive changes were also observed in the left *(t* = −2.272, *p* < 0.05) and right (*t* = −2.475, *p* < 0.05) brains on ATQ. However, no statistically significant changes were observed in the control group.

In addition, the stress scale of the experimental group showed a significant change in all sub-categories: parents (*t* = 2.226, *p* < 0.05), family (*t* = 2.941, *p* < 0.05), friends (*t* = 2.460, *p* < 0.05), study (*t* = 2.609, *p* < 0.05), and school (*t* = 2.676, *p* < 0.05). Among the sub-categories of self-esteem, there were statistically significant results in overall self-esteem and social self-esteem (*t* = −5.140, *p* < 0.05 and *t* = −2.629, *p* < 0.05, respectively). However, among the sub-items of self-esteem, there were no statistically significant results regarding self-esteem at home and self-esteem in school (*t* = −1.967, *p* < 0.05 and *t* = 0.558, *p* < 0.05, respectively); in the control group, there were no significant results in any of the scales.

## 4. Discussion

This study aimed to investigate how nature-based group art therapy affected brain waves, stress, and self-esteem in non-disabled siblings of children with disabilities. The main results analyzed in the study are discussed as follows.

First, nature-based group art therapy has brought positive changes to EEG. The ATQ is related to theta (θ) waves and SMR waves and creativity in the setting of alpha (α)-theta (θ) crossover. Theta (θ) waves are involved in the encoding and retrieval of working memory, and they play an important role in peak performance. Activity in the forest increased the number of alpha (α) waves generated, leading to a crossover with theta (θ) waves. SMR waves are closely related to concentration, corresponding to the arousal preparation state or the motor system’s standby state [49,50]. These waves only appear in the sensory domain and are related to immunity; thus, they can lead to positive physical changes.

High ATQ indicates that the brain is alert and that immune function is high. After the eighth session in the present study [45], positive changes were observed in the left and right hemispheres. Additionally, high ATQ leads to steadier nerves, increased concentration, and decreased fatigue [51], indicating a correlation between ATQ and ASQ.

ASQ is calculated based on the correlation between delta (δ) waves and high beta (β) waves, which are the 21–30 Hz waves that appear during a tense, excited, or stressful state. Low ASQ leads to higher fatigue and lower disease resistance, while high ASQ leads to greater disease resistance [44]. As a result, nature-based art therapy increased the participant’s ability to resist stress. At the same time, positive ASQ changes indicate that stress is relieved and the immune system is further strengthened.

Furthermore, nature-based group art therapy facilitated stress reduction. The pre-and post-test results of the experimental group showed that the total stress score significantly decreased, as did the scores in all sub-categories (parents, family, friends, academics, and school). However, there were no significant differences between the pre-and post-test scores of the control group. The causes and consequences of stress lower hippocampal function, resulting in the loss of neuroplasticity and long-term neurobiological disorders, which can negatively affect the ability to learn and change [52].

Finally, nature-based group art therapy had a positive effect on self-esteem. High self-esteem means that one gives high value to oneself, recognizes one’s strengths, has positive feelings, and has high confidence in daily life. The experimental group’s pre-and post-test results showed that total self-esteem scores and scores of the sub-categories, such as overall self-esteem and social self-esteem, increased significantly after the program. The average score of self-esteem at home positively changed, but it was not statistically significant. Additionally, their self-esteem at school did not show a significant change. In contrast, the control group results showed no significant differences between the pre-and post-tests.

### Limitations and Suggestions for Future Research

The limitations of this study and suggestions for future research are as follows.

First, the study was conducted with a relatively small sample size. The dropout rate in the control group was high, resulting in a difference in the number of the control group and the experimental group. This may impair the generalization of potential of the study. Second, only non-disabled siblings of children with disabilities accompanied by their parents were allowed to participate. The difficultly for parents to accompany participants twice weekly to the site limited the recruitment of more participants. Third, this study did not consider the influence of environmental factors, such as the function of the family of participants or the difference in the surrounding environment. Finally, there is a limit to regional generalization because the research participants were not recruited from various regions.

As a recommendation, it is necessary to confirm the effectiveness of nature-based art therapy through a broader range of study participants in future studies. The changes in the body can also be studied using different psychophysiological protocols. The support system of the home or school, or other social factors, can also influence the outcomes; therefore, an appropriate experimental design will be required to control for such variables.

## 5. Conclusions

Nature-based group art therapy helps activate and implant experiences that stimulate senses in the brain, such as visual, auditory, tactile, olfactory, and taste encounters, through various memory pathways. In addition, experiencing a new environment in nature can broaden thought and experiences through independent observation and exploration of materials.

The above results show that nature-based group art therapy has a positive effect on non-disabled children by increasing their resistance to disease, alleviating stress, and increasing self-esteem.

## Figures and Tables

**Table 1:** Group art therapy programs.

Scheme.	Program	Expected Effect	Activities
1	Feeling and expressing the five senses	To relieve stress and tension	Feeling nature’s sounds, smells, touch, and light, and freely expressing these feelings
2	Leaf frottage	To relieve stress and strengthen attention span	Observing and expressing various leaves in nature; making a wish tree
3	Natural mandala	To strengthen psychological stability and concentration	Forming a collective mandala using various natural objects
4	Making colored sand	For psychological relaxation and to strengthen concentration	Drawing a picture using various sands surrounding them
5	Making a map— forest explorer	To relieve stress and strengthen attention span	Making a map of the forest in pairs
6	Photo therapy	To strengthen attention span and concentration	Taking pictures of nature and drawing favorite parts
7	Making a hideout	To relieve stress and improve social skills	Making a house using cloth and wood with the group members
8	Molding natural objects and photos	To relieve stress and improve sense of achievement	Creating your own sculpture using your own photos, branches, leaves, grass, and flowers

**Table 2:** The brain function quotient.

Analyzing Quotient	Hemisphere	Related Frequency	Characteristics
Attention quotient (ATQ)	Left, right	Theta (θ) wave SMR	Decision of brain awakening degree, resistance to disease or physical fatigue
Anti-stress quotient (ASQ)	Left, right	Delta (δ) wave High beta (β) wave	Decision of physical, mental stress resistance degree

**Table 3:** Participants’ characteristics and work.

Classification		Frequency (Subject Number) (*n* = 29)	Percentage (%)
Gender	Male	18	62.1
	Female	11	37.9
Age	7 years old	8	27.6
	8 years old	10	34.5
	9 years old	7	24.1
	12 years old	2	6.9
	13 years old	2	6.9
All		29	100.0

**Table 4:** Group homogeneity test.

Classification	Control Group (*n* = 18)	Experimental Group (*n* = 11)	*t*	*p*
	*M*(*SD)*	*M(SD)*		
Gender	1.39 (0.5016)	1.36 (0.505)	0.131	0.897 *
Age	9.17 (0.7859)	10.27 (2.6112)	−1.368	0.198 *
Stress	72.11 (26.451)	75.45 (20.806)	−0.356	0.724 *
Self-esteem	109.11 (13.425)	106.09 (9.782)	0.647	0.523 *

* *p >* 0.05.

**Table 5:** Changes in the ATQ, ASQ, stress, and self-esteem scales of the experimental and control groups.

Classification		Control Group (*n* = 15) *M (SD)*				Experimental Group (*n* = 20) *M (SD)*			
		Before	After	*t*	*p*	Before	After	*t*	*p*
ATQ	Left	58.74 (8.268)	51.92 (10.191)	1.802	0.102	62.41 (9.892)	66.62 (7.59)	−2.741	0.014 *
	Right	63.07 (7.254)	56.95 (10.956)	1.343	0.209	67.78 (7.72)	67.73 (7.77)	−2.272	0.036 *
ASQ	Left	65.76 (19.504)	53.90 (22.273)	1.004	0.339	62.22 (15.48)	67.97 (14.49)	−2.256	0.038 *
	Right	69.44 (8.023)	57.12 (23.055)	1.741	0.112	62.16 (117.64)	70.51 (16.61)	−2.475	0.025 *
Stress scale	Parents	16.09 (4.547)	16.72 (7.824)	−0.322	0.754	16.22 (5.897)	12.72 (4.336)	2.226	0.040 *
	Family	13.72 (4.941)	15.72 (6.915)	−1.736	0.113	13.72 (5.49)	9.11 (2.72)	2.941	0.009 *
	Friends	14.09 (6.347)	13.45 (6.072)	0.533	0.605	13.11 (6.19)	8.89 (2.63)	2.460	0.025 *
	Study	18.55 (5.646)	17 (5.709)	2.152	0.057	15.17 (5.26)	11.67 (5.30)	2.609	0.018 *
	School	13 (3.975)	13.27 (5.178)	−0.289	0.779	13.72 (7.00)	8.89 (2.65)	2.676	0.016 *
	Total score	75.45 (20.806)	76.18 (28.979)	−0.159	0.877	71.94 (26.55)	51.28 (13.99)	3.002	0.008 *
Self-esteem scale	Overall self-esteem	18 (3.41)	18.18 (4.51)	−0.132	0.897	17 (2.99)	20.56 (3.28)	−5.140	0.000 *
	Social self-esteem	31 (5.78)	28.82 (6.74)	1.544	0.154	27.89 (6.14)	32.33 (6.19)	−2.629	0.018 *
	Self-esteem at home	32 (7.97)	33 (8.69)	−0.489	0.635	35.17 (7.13)	38.44 (4.92)	−1.967	0.066
	Self-esteem in school	20.72 (4.24)	21.36 (4.61)	−0.449	0.663	22.78 (7.37)	21.78 (5.23)	0.558	0.584
	Total score	106.09 (9.78)	105.36 (20.37)	0.190	0.853	107.00 (16.56)	117.67 (12.18)	−2.424	0.027 *

ATQ, attention quotient; ASQ, anti-stress quotient. * *p <* 0.05.

## Data Availability

The data presented in this study are available on request from the corresponding author. The data are not publicly available to protect confidentiality of the research participants.

## Supplementary Materials

The following are available online at https://www.mdpi.com/article/10.3390/ijerph18115912/s1, Table S1: Group art therapy programs.
