# Supplementary material for: Effects of Nature-Based Group Art Therapy Programs on Stress, Self-Esteem and Changes in Electroencephalogram (EEG) in Non-Disabled Siblings of Children with Disabilities
Source: Int J Environ Res Public Health. 2021 May 31;18(11):5912. doi: 10.3390/ijerph18115912 (PMC8199280; doi:10.3390/ijerph18115912)
Supplement: Supplementary file 1 [file ijerph-18-05912-s001.zip › ijerph-1183046-supplementary.pdf]

Supplementary Material

Table S1. Group art therapy programs.

Session	Program	Expected Effect	Activities
1	Introducing myself	Pre-evaluation and inner search	Introduce yourself while appreciating nature
2	Feeling and expressing the five senses	To relieve stress and tension	Feeling nature's sounds, smells, touch, and light, and freely expressing these feelings
3	Leaf frottage	To relieve stress and strengthen attention span	Observing and expressing various leaves in nature; making a wish tree
4	Natural mandala	To strengthen psychological stability and concentration	Forming a collective mandala using various natural objects
5	Making colored sand	For psychological relaxation and to strengthen concentration	Drawing a picture using various sands surrounding them
6	Story about trees	Stress relief and attention-getter	Talk about different types of trees and decorate tree rings
7	Making a fruit	Stress relief and improved sociality	Observe trees and make your own fruits
8	Drawing using sand	Stress relief and psychological relaxation	Draw things using different types of sand
9	Making a map -Forest explorer	To relieve stress and strengthen attention span	Making a map of the forest in pairs
10	Expression using stones	Stress relief and stability	Make and design your own comfortable space using various types of stones
11	Story about charcoal	Stress relief and increased sense of accomplishment	Expressing nature while understanding and using charcoal
12	Photo therapy	To strengthen attention span and concentration	Taking pictures of nature and drawing favorite parts
13	Making a hideout 1	To relieve stress and improve social skills	Making a house using cloth and wood with the group members
14	Making a hideout 2		
15	Molding natural objects and photos	To relieve stress and improve sense of achievement	Creating your own sculpture using your own photos, branches, leaves, grass, and flowers
16	I think the forest is…	Stress relief and positive self-image	Illustrate your meaning of the forest on a postcard and decorate it with other group members.
